# Ultrasound-Assisted Polysaccharide Extraction from Grape Skin and Assessment of In Vitro Hypoglycemic Activity of Polysaccharides

**DOI:** 10.3390/foods14101801

**Published:** 2025-05-19

**Authors:** Wei Li, Na Wang, Ting Xu, Qingping Du, Rui Yang, Mingxun Ai, Xinyao Han, Wei Wang

**Affiliations:** College of Food Science and Pharmacy, Xinjiang Agricultural University, Urumqi 830052, China; liweili2025@163.com (W.L.);

**Keywords:** grape skin, polysaccharide, extraction process, hypoglycemic activity

## Abstract

Grapes are commonly processed into shelf-stable products such as raisins, wine, juice, and syrup-canned syrup goods. During processing, byproducts like skins and seeds are generated, which contain bioactive compounds including polysaccharides and polyphenols that exhibit diverse biological activities. The objective of this work was to thoroughly evaluate the impact of ultrasound technology on both the extraction efficiency and in vitro hypoglycemic activity of the polysaccharides derived from grape skin. The isolation and purification of the polysaccharides were carried out using chromatographic column techniques, and the monosaccharide components were determined through HPLC. The hypoglycemic activity of the polysaccharides from grape skin in vitro was analyzed in vitro considering their inhibitory effects on α-amylase and α-glucosidase. The polysaccharides from grape skins were extracted via an ultrasound-assisted methodology (under the following conditions: 50 °C, 50 min, 20 mL/g ratio, and 210 W), resulting in an 11.82% extraction yield of GSPs. Monosaccharide constituent analysis revealed that GSP-1-1 consisted of galacturonic acid, arabinose, rhamnose, galactose, glucose, glucuronic acid, mannose, and xylose in a molar ratio of 40.26:26.99:13.58:12.2:2.24:1.97:1.63:1.42. In vitro evaluations indicated that both GSP and GSP-1-1 exhibited notable suppression of α-amylase and α-glucosidase activities, two key enzymes in carbohydrate digestion. This dual inhibitory action positions these compounds as potential therapeutic agents for blood glucose management strategies. This work provides a new direction for addressing the byproducts of the grape canning industry and also offers a theoretical basis for the development of functional grape products.

## 1. Introduction

The increase in the generation of industrial waste generation has become the main problem facing food processing operations. Approximately 39% of the global waste is produced by industrial activities is approximately 39% [1]. The valorization of process-derived byproducts demonstrates substantial market viability and the potential for economic returns [2]. Grapes are among the oldest and most widely produced fruits in the world [3]. They are characterized by their succulent texture and distinctive aromatic profile, containing abundant nutritional components including phenols, oils, proteins, vitamins, organic acids, and minerals [4,5]. Grapes are commonly processed into raisins, wine, juice, and syrup-packed canned products for preservation due to their lack of perishability on account of their elevated water content and sugar levels [6]. The canning process typically involves manual or alkaline peeling, generating organic byproducts (peels and seeds), which are often discarded or repurposed as fertilizer through fermentation. This practice not only undermines environmental sustainability but also represents significant resource underutilization.

Grape skin contains water-soluble polysaccharides, which create new possibilities for integrated utilization [6,7]. As naturally abundant biomacromolecules, polysaccharides have many different properties and critical biological functions across various living organisms [8,9]. For instance, they possess antioxidant, immunomodulatory, antitumor, antihypertension, anti-inflammatory, antibacterial, and antidiabetic effects [10,11,12,13,14,15]. It was shown that polysaccharides from Beichun and Benni fuji exhibited potent antioxidant functions and ameliorated myocardial ischemia–reperfusion (I/R) injury in murine models. Cabernet Sauvignon-derived polysaccharides demonstrated multi-organ (pulmonary/hepatic/renal) protective effects in murine polymicrobial sepsis models through a significant attenuation of systemic inflammatory responses [16].

Given the importance of the polysaccharides in grape skin, it is extremely crucial to understand their properties and biological activities. Polysaccharide extraction methods are closely related to the yield and structural integrity of polysaccharides [17,18,19]. Traditional polysaccharide extraction methods have limitations such as their low efficiency and time-consuming processes. On the other hand, the ultrasound-assisted extraction method can promote the dissolution of polysaccharides by destroying the plant cell wall, and offers advantages such as low cost and easy industrial scalability [18,20]. Therefore, this study aimed to extract and isolate polysaccharides from grape skin (GSPs) and investigate their properties, encompassing monosaccharide composition, molecular weight distribution, and hypoglycemic activity, in vitro. This work establishes a theoretical basis for the resource development of GSPs.

## 2. Materials and Methods

### 2.1. Materials and Reagents

The grape skin of the *Muscat hamburg* variety, collected from Bei Yuan Chun Market (Urumqi, Xinjiang, China), was dried at 50 °C and ground into powder that was sieved through a 60 mesh sieve. DEAE-52 and Sephadex G-100 resins and dialysis membranes (molecular weight cut-off: 8–14 kDa) were purchased from Solarbio Technology Co., Ltd. (Beijing, China). The activities of α-amylase and α-glucosidase were determined using commercial assay kits from Solarbio (Beijing, China). Sodium nitrate, trifluoroacetic acid (TFA), and trifluoroacetic acid were purchased form Shanghai Anpel Experimental Technology Co., Ltd. (Shanghai, China). Sodium acetate, potassium bromide, and monosaccharide reference standards were obtained from Sigma-Aldrich (St. Louis, MO, USA).

### 2.2. The Extraction and Fractionation of Polysaccharides from Grape Skin

#### 2.2.1. Single-Factor Experiments for GSP Extraction

Ultrasound equipment (KQ-300DE) was bought from Kun Shan Ultrosonic Instruments Co., Ltd. (Kunshan, China). The grape skin powder was effectively cleared of unwanted impurities and extracted with distilled water under an ultrasonic power of 180 to 270 W, an ultrasonic time of 30 to 70 min, a temperature of 30 to 70 °C, and a liquid-to-solid ratio of 10 to 30 mL/g. All tested samples underwent more than three parallel experiments. Following condensation to 1/8 volume via rotary evaporation, the solution was treated with threefold anhydrous ethanol (*v*/*v*) at 4 °C for 12 h to induce precipitation, centrifuged at 8000 rpm for 5 min, and dried at 50 °C. The protein was repeatedly removed using the Sevag reagent [8,21], followed by treatment with petroleum ether. Finally, the extraction yield of GSPs was determined gravimetrically using the following formula:

(1) $$(\%)Yield=\frac{C}{W}\times 100$$

where C represents the GSP weight (g), and W denotes the sample powder weight (g).

#### 2.2.2. Box–Behnken Design and Statistical Evaluation

RSM-BBD optimization revealed ultrasonic time (Z_1_), ultrasonic temperature (Z_2_), and the liquid-to-solid ratio (Z_3_) as critical parameters for enhancing GSP yield, initially screened via monofactorial trials. Each factor was assigned to three levels, coded as +1, 0, and −1 to represent high, central, and low actual values, respectively (Table 1). The statistical optimization of GSP extraction was performed using a 17-run BBD. Stat-Ease Design Expert (Version 13.0.1.0, Stat-Ease, Inc., Minneapolis, MN, USA) was utilized to generate and assess the results (Table 2). All tested samples underwent more than three parallel experiments.

#### 2.2.3. Purification of GSP

We subjected 5 mL of GSP solution (50 mg/mL) was subjected to a DEAE Fast Flow column, which had previously reached equilibrium with 200 mL of Tris buffer (20 mM, pH 8.0). The column was subsequently eluted using isocratic conditions with buffer and with sequentially increasing NaCl concentrations (0 → 0.1 → 0.3 → 0.5 mol/L) at a 1.0 mL/min flow rate. Eluate fractions (10 mL/tube) were automatically collected and sequentially processed through vacuum concentration, membrane dialysis, and freeze-drying. The resulting product was chromatographed on a Sephadex G-100 column using deionized water as the mobile phase at a 0.2 mL/min flow rate. The polysaccharide content was determined using the phenol-sulfuric acid method [3].

### 2.3. The Properties of the GSP Fraction

#### 2.3.1. Molecular Weight (M_W_) Distribution

Based on a previously reported approach [22,23], the molecular weight characterization was performed using HPLC (high-performance liquid chromatography, Thermo UltiMate3000, Thermo Fisher Scientific, Waltham, MA, USA). The analysis was conducted in 0.1 M sodium nitrate aqueous solution containing 0.02% sodium azide at 45 °C. Mobile-phase delivery was maintained at a 0.6 mL/min flow rate throughout the experiment. Concurrent detection was achieved for the simultaneous determination of the eluent concentration and refractive index increment. The experimentally derived specific refractive index increment (dn/dc) value for the polymer solution under these analytical conditions was established as 0.141 mL/g. This dual-detector configuration enabled the comprehensive characterization of macromolecular parameters through complementary light-scattering and refractive index measurements.

#### 2.3.2. Chemical Composition of GSPs

The total sugar content present in the GSPs was determined following the phenol-sulfuric acid method using glucose as the standard [3]. The protein content in the GSPs was measured by the Bradford method, using bovine serum albumin serving as the standard [24]. The total uronic acid and sulfate groups in the GSPs were determined by using the m-hydroxybiphenyl method and barium chloride-gelatin method, respectively [25]. We accurately weighed 10 mg of the sample to prepare a 10 mg/L solution for the experiment, and all tested samples were set up in more than three parallel experiments.

#### 2.3.3. Monosaccharide Profiling of GSPs

The monosaccharide profiling of GSPs was adapted from a method reported in the literature and slightly modified [26,27]. Briefly, 20 mg of powder was hydrolyzed in 1 mL of TFA (2.0 M) at 121 °C for 2 h in a sealed glass tube, which was purged with nitrogen gas and blow-dried. We added 99.99% methanol for cleaning, and then blow-dried the sample again. We repeated the methanol cleaning process 2 to 3 times. The sample was reconstituted in sterile water and analyzed via ion chromatography (Thermo ICS 5000+, Thermo Fisher Scientific, Waltham, MA, USA), equipped with electrochemical detection. For chromatographic separation, we utilized a Dionex CarboPac PA20 (Thermo Fisher Scientific, Waltham, MA, USA) column with a ternary mobile phase: H₂O (A), 0.1 M NaOH (B), and a mixture of 0.1 M NaOH/0.2 M NaAc (C). These were delivered at a flow rate of 0.5 mL/min at 30 °C, with an injection volume of 5 μL. The gradient elution was performed according to the schedule below: at 0 min (95:5:0, A/B/C, *v*/*v*); at 26 min (85:5:10); at 42 min (85:5:10); at 42.1 min (60:0:40); at 52 min (60:40:0); at 52.1 min (95:5:0). This composition was then held constant until 60 min. The analysis involved thirteen reference standards, with chromatographic conditions identical to those described in the preceding section. Data were acquired on an ICS5000+ (Thermo Fisher Scientific, Waltham, MA, USA) and processed using chromeleon 7.2 CDS (Thermo Fisher Scientific, Waltham, MA, USA).

#### 2.3.4. FT-IR

The FT-IR spectra of the GSPs were acquired using a Nicolet iZ-10 spectrometer (Thermo Scientific, Waltham, MA, USA). The polysaccharide samples were mixed with KBr powder and then pressed into 1 mm pellets for FT-IR measurement with a Smart iTR (Thermo Nicolet, Waltham, MA, USA) in 4000–450 cm^−1^.

#### 2.3.5. UV Spectroscopy

The GSPs were measured and formulated into a 0.5 mg/mL polysaccharide solution. The ultraviolet spectrum characteristics of the GSPs were characterized by full-wavelength scanning (200–600 nm) using a ultraviolet spectrophotometer (Shanghai Mepuda Instrument Co., Ltd., Shanghai, China).

#### 2.3.6. Scanning Electron Microscopy

For SEM analysis, the GSPs powder were covered with a thin layer of gold and subsequently examined under a scanning electron microscope (MERLIN Compact, Zeiss Merlin Compact, Oberkochen, Germany) at 1.0 kV, using magnifications of 500- and 10,000-fold magnifications [8].

### 2.4. The Anti-α-Amylase Activity

A previously report method was adapted from reported method with some alterations [28]. Briefly, 100 µL of α-amylase solution (1.0 U/mL, in 20 mM potassium phosphate buffer, pH 6.9) was mixed with equal volumes of sample solutions at different concentrations and mixed thoroughly. The mixed sample solutions were incubated at 37 °C for 10 min. Thereafter, 100 µL of a 1% starch solution was added to the reaction mixture, followed by thorough mixing. The well-mixed reaction solution was then incubated at 37 °C for an additional 10 min. After that, we added 750 µL of 3,5-dinitrosalicylic acid to the resulting solution a to terminate the reaction. The combined solution was heated in a boiling water bath for 10 min, after which the absorbance was recorded at 540 nm, with acarbose serving as the positive control, all tested samples were set up with three parallel experiments, the percentage inhibition was determined applying the equation:

(2) %inhibitoin=[1−(A−B)C]×100

where A was the test sample with polysaccharides, B was the enzyme-free control, and C was the polysaccharide-free blank. Freshly prepared α-amylase and starch solutions were utilized within 24 h of preparation.

### 2.5. The Inhibitory Effect on α-Glucosidase Activity

The suppressive impacts of GSPs on α-glucosidase activity were evaluated through an adapted version of a previously described technique [29]. In brief, 40 µL of 0.5 U/mL α-glucosidase solution (in 0.1 M potassium phosphate buffer, pH 6.9) was individually mixed with 20 µL of polysaccharide solution at graded concentrations. Following a 10 min pre-equilibration period at 37 °C, enzymatic reactions were initiated by introducing 100 µL of 5 mM pNPG solution to all test systems. Subsequent substrate hydrolysis proceeded for 20 min under controlled thermal conditions. The reaction was terminated using 100 µL of 0.1 M sodium carbonate, with subsequent spectrophotometric quantification performed at 405 nm, with acarbose serving as the positive control. All tested samples were set up in three parallel experiments. The percentage inhibition was determined by utilizing the following equation:

(3) %inhibition=(1−A/B)×100

The absorbance parameters were defined as follows: A was the test group containing GSP, GSP-1-1, or acarbose; B was the blank control.

### 2.6. Statistical Analysis

Date were statistically analyzed using SPSS 26.0 (IBM Corp., Armonk, New York, NY, USA) and are presented as mean ± standard deviation from three independent replicates. Intergroup differences were analyzed using one-way ANOVA, followed by Duncan’s multiple range test. Statistical significance was set at *p* < 0.05. All graphical representations were generated with OriginPro 2021 (OriginLab Corporation, Northampton, MA, USA).

## 3. Results and Discussion

### 3.1. Analysis of Results of the Single-Factor Test

**Figure 1:**
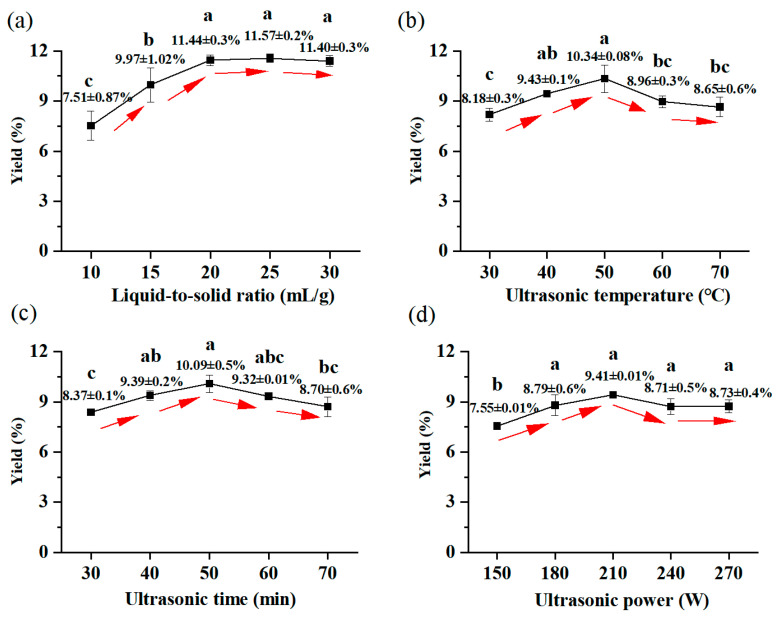
The effect of liquid-to-solid ratio on GSP yield: (**a**) ultrasonic temperature, (**b**) ultrasonic time, (**c**) ultrasonic power. (**d**) The direction of the arrow indicates the direction of the trend. Distinct superscript letters (a–c) within columns denote statistical significance (*p* < 0.05).

In this single-factor experiment, this study focused on selecting and examining several critical parameters: ultrasonic temperature, ultrasonic duration, liquid-to-solid ratio, and ultrasonic power. The liquid-to-solid ratio was determined to play a significant role in impacting the yield of polysaccharides [30]. As exhibited in Figure 1a, the yield of GSPs increased rapidly from 7.51% to 11.44% as the liquid-to-solid ratio rose from 10 to 20 mL/g, and subsequently stabilized at 11.40% when the ratio reached 30 mL/g. Ultrasonic temperature was another critical factor affecting extraction efficiency [31]. Figure 1b demonstrated that the extraction rate initially rose with the increase in temperature, reaching its peak at 10.33% when the ultrasonic temperature was at 50 °C, and then progressively decreasing at higher temperatures. According to Figure 1c, there was a same trend as for ultrasonic temperature: as time increased to 50 min, the yield rate also increased correspondingly to 10.08%. After more than 50 min, the yield rate decreased. So, 50 min extraction was found to be the optimal for extraction temperature. As depicted in Figure 1d, the highest GSP yield of 9.41% was achieved with an ultrasound input power of 210 W. Sonication energy has been demonstrated to induce progressive cell wall disruption through cavitation effects, with polysaccharide liberation efficiency being positively correlated to the applied intensity during the initial extraction stages [32,33]. Beyond the optimal sonication parameters, elevated energy densities induce oxidative cleavage of polysaccharide chains through free radical generation, ultimately compromising extraction efficiency due to molecular fragmentation [34], so 210 W was the best choice from the perspective of energy savings.

### 3.2. Optimization of Extraction Conditions Using RSM

In accordance with the initial experiments and the Box–Behnken design (BBD) approach, three separate factors (Z_1_, Z_2_, and Z_3_) were assigned coded levels of −1, 0, and +1. Using Design Expert 13.0 (Stat-Ease, Minneapolis, MN, USA), a response surface analysis comprising 17 experimental runs was systematically designed. The design layout and corresponding outcomes are displayed in Table 2.

The BBD experimental results were statistically evaluated through Design Expert 13.0 software, with polysaccharide extraction efficiency assigned as the dependent variable (Y). The regression model equation was expressed as:

Y = − 340.598 + 5.99322A + 7.41342B + 1.4027C − 0.01067AB + 0.01014AC + 0.0027BC − 0.056255A2 − 0.068755B2 − 0.048965C2(4)

**Figure 2:**
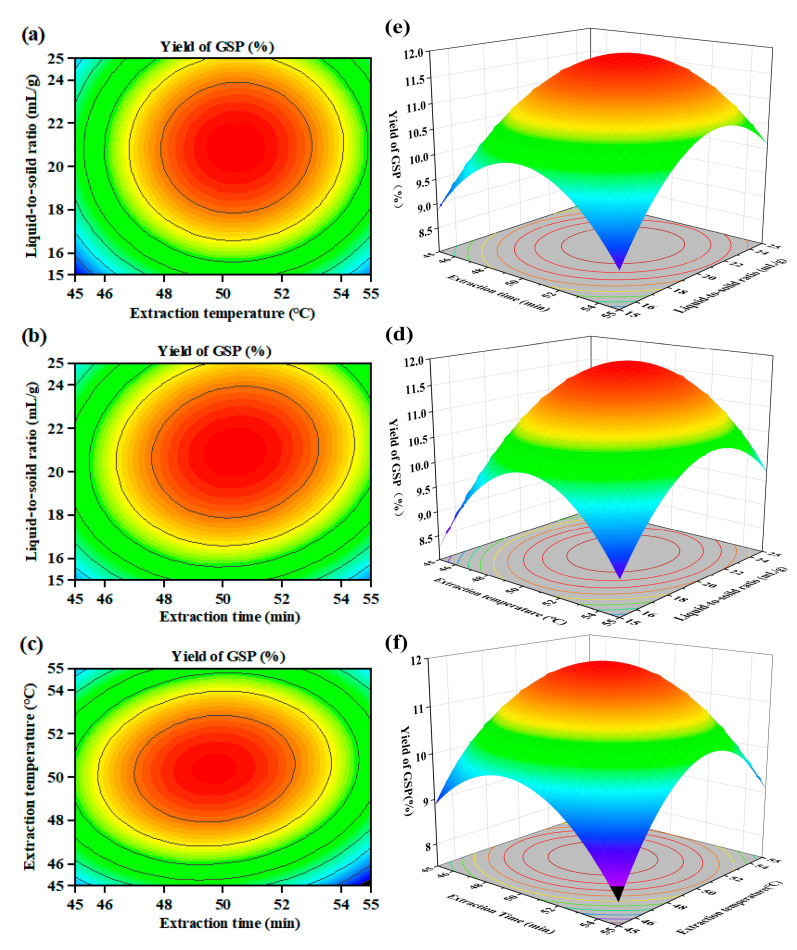
The 2D contour plots (**a**–**c**) and 3D response surface plots (**d**–**f**) of GSPs. The redder the color, the higher the yield.

In this regression model equation, all quadratic coefficients (Z_1_^2^, Z_2_^2^, Z_3_^2^) are negative, so the equation has the maximum value. The response values from the BBD experiments and the ANOVA results of the regression model are presented in Table 3 respectively. Based on the F-statistic analysis (Z_1_Z_2_ (F = 4.810) > Z_1_Z_3_ (F = 4.340) > Z_2_Z_3_ (F = 0.308)), the hierarchical influence of the factor interactions on the yield was quantified as: ultrasonic time × ultrasonic temperature > ultrasonic time × liquid-to-solid ratio > ultrasonic time × liquid-to-solid ratio. The 3D response surface plots illustrating factor interactions are presented in Figure 2. The ideal extraction conditions for GSPs were identified as a 50.4423 °C of ultrasonic temperature, a 20.8909 mL/g of liquid-to-solid ratio, and a 50 min of extraction time. During the experiment, the temperature exhibited progressive elevation as the ultrasonic time increased. It was difficult to control the temperature to the decimal place, and measuring the liquid-to-solid ratio to such precision proved unnecessary. Therefore, the liquid-to-solid ratio was impossible to verify according to the optimal simulated process. Instead, a close achievable index was selected for verification (liquid-to-solid ratio, 20 mL/g; extraction temperature, 50 °C; extraction time, 50 min; extraction power, 210 W). The experimentally measured extraction yield of 11.92 ± 0.44 mg/g demonstrated strong agreement with the model-predicted value (11.89 mg/g), which confirmed the appropriateness of the model equation. During industrial production, the temperature usually fluctuates during the production time; therefore, it is difficult to maintain it at a precise temperature. Achieving production parameters accurate to the decimal point usually require high-end instruments, which increase the production costs. To facilitate actual operation, the optimized parameters were established: liquid-to-solid ratio, 20 mL/g; extraction temperature, 50 °C; extraction time, 50 min; extraction power, 210 W.

### 3.3. Chemical Composition of GSP

The contents of total sugar, protein, uronic acid, and sulfate in the GSPs are shown in Table 4. As presented in the table, the purification process significantly affected the total sugar and protein contents in the purified fractions. Following purification, there was a notable increase in the total sugar and uronic acid contents of GSP-1-1, reaching 87.79 ± 0.51% and 41.66 ± 0.10%, respectively, whereas the protein content became undetectable. Additionally, the purification process effectively decreased the sulfate content in crude GSPs, correlating with the sulfation reduction pattern documented in analogous polysaccharide studies [35].

### 3.4. Elution Curve and Molecular Weight of GSPs

**Figure 3:**
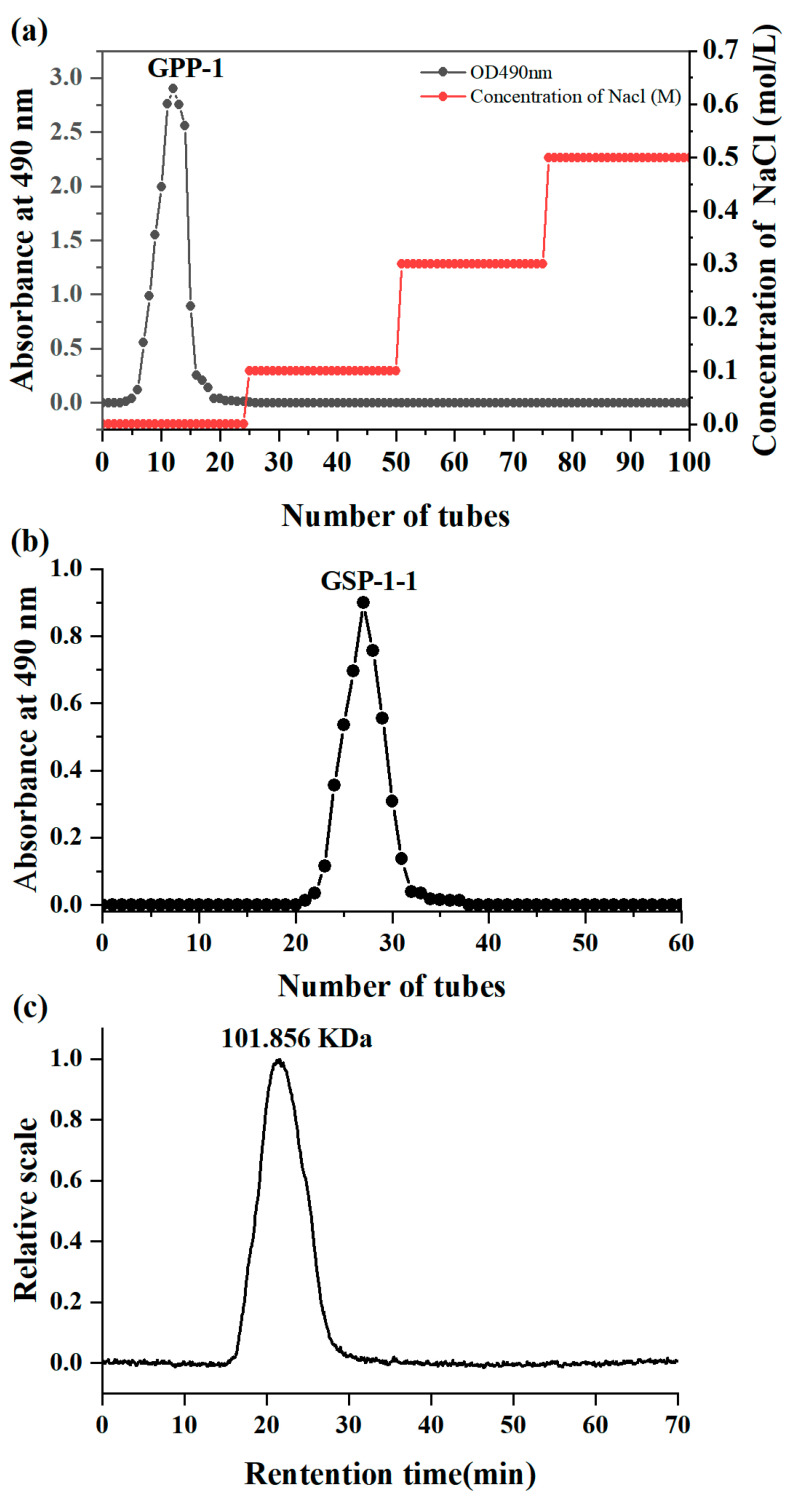
Elution curve of crude GSPs on DEAE-52 cellulose chromatography column, (**a**). Elution curve of crude GSP-1 on cellulose chromatography column, (**b**). Molecular weight results for GSP-1-1, (**c**).

Figure 3a,b show that the purification protocol involved sequential separation processes: anion exchange (DEAE-52 cellulose) followed by molecular sieving (Sephadex G-100). The target fraction underwent vacuum concentration, dialysis, and lyophilization, which was named as GSP-1-1 with a moisture content of 1.65 ± 0.12%.

The molecular weight of polysaccharides is critically linked to their absorption into the human body and functional efficacy [36]. Generally, homopolysaccharides with molecular weights ranging between 10 and 200 kDa exhibit higher bioactivity. Figure 3c exhibits a sharp monodisperse peak in the molecular weight analysis, indicating that the purified polysaccharide was homogeneous [37], which had a Mw of 101.856 kDa. The polydispersity index of GSP-1-1 was measured as 1.620, and a moderate degree of polydispersity maximizes the functional activities of polysaccharides [32].

### 3.5. FT-IR Analysis and UV Spectroscopy

**Figure 4:**
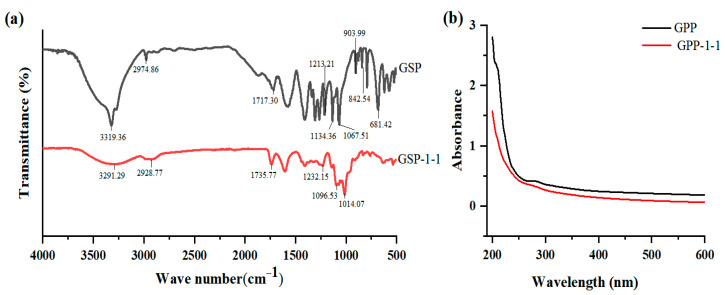
FT-IR spectra of GSPs (**a**) and UV spectroscopy of GSPs (**b**).

The infrared spectral analysis in Figure 4a reveals that the absorption peaks observed in the 3200–3500 cm^−1^ range (GSP: 3319.36 cm^−1^; GSP-1-1: 3291.29 cm^−1^) can be attributed to O-H stretching vibrations. The minor absorption bands detected in the 2850–2960 cm⁻^1^ range (GSP: 2974.86 cm^−1^; GSP-1-1: 2928.77 cm^−1^) correspond to C-H stretching vibrations [38,39]. The characteristic peaks appearing in the 1700–1750 cm^−1^ range (GSP: 1717.30 cm^−1^; GSP-1-1: 1735.77 cm^−1^) arise from the asymmetric stretching vibrations of the C=O group. Furthermore, the peak observed at 1604.89 cm^−1^ originated from asymmetric stretching vibrational modes, which provided characteristic spectral evidence for uronic acid constituents within the polysaccharide fraction of GSP-1-1 [28,30]. In addition, the absorption features observed at 903.99 cm^−1^ and 681.42 cm^−1^ for the GSPs could be assigned to pyridine ring stretching vibrations. Notably, the absorption peak at 842.54 cm⁻^1^ was indicative of α-glycosidic configurations in the GSPs [35,40]. Additionally, the region spanning 1000–1200 cm^−1^ is recognized as the fingerprint zone for polysaccharides, with characteristic absorptions attributable to C-O-C and C-O-H bond-stretching vibrations [41,42], complemented by detectable O-H group deformations in this critical identification band. The absorption peaks detected at 1067.51 cm^−1^ 1134.36 cm^−1^, and 1213.21 cm^−1^ for GSP, and 1014.07 cm^−1^, 1096.53 cm^−1^, and 1232.15 cm^−1^ for GSP-1-1, respectively, further corroborate the polysaccharide structures of both GSP-1-1 and GSP.

The purities of the GSP and GSP-1-1 were determined by UV spectroscopy at 490 nm. The UV spectrum of GSP (Figure 4b) revealed a hypochromic shift pattern, with a faint absorption signature observed at 260–280 nm, suggestive of proteins or nucleic acid constituents within the polysaccharide. The UV spectrum of GSP-1-1 decreased smoothly with almost no absorption peak, indicating that the proteins were removed completely during the purification process [32,35].

### 3.6. HPLC Analysis of Monosaccharide Composition

**Figure 5:**
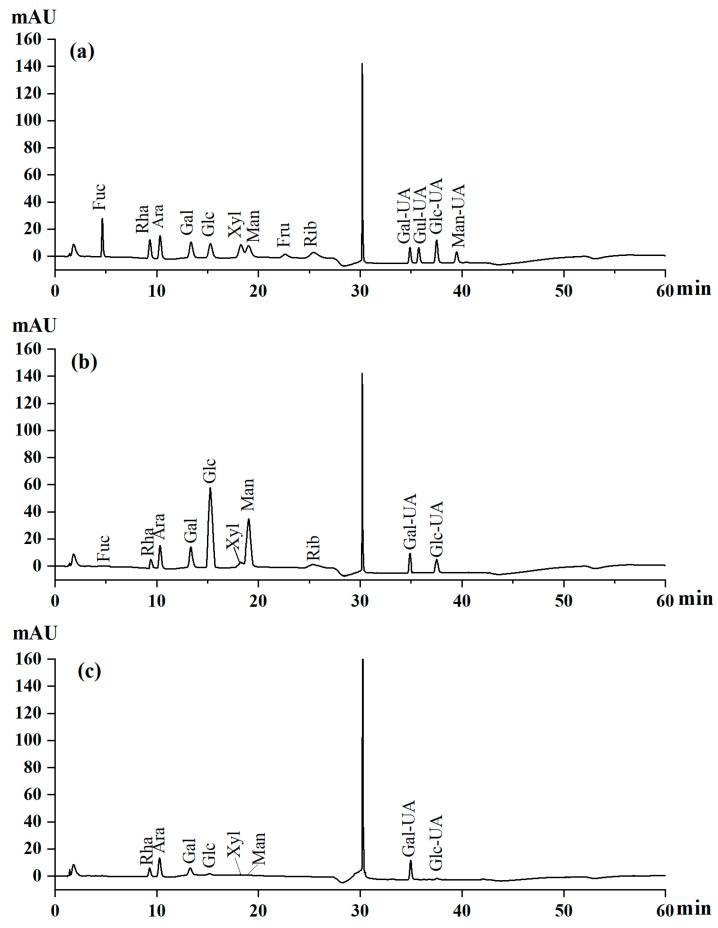
Monosaccharide composition analysis of standards (**a**). monosaccharide composition analysis of GSP (**b**). monosaccharide composition analysis of GSP-1-1 (**c**).

Comparing the chromatograms of the 13 standard mixed solutions (Figure 5a), 10 different monosaccharides were found in the GSPs (Figure 5b). They predominantly consisted of glucose, mannose, arabinose, and galactose, accounting for 43.80%, 14.43%, 14.32%, and 11.82%, respectively. Small amounts of glucuronic acid, galacturonic acid, rhamnose, xylose, ribose, and fucose accounted for 6.36%, 3.15%, 2.56%, 1.36%, 0.56%, and 0.38%. Meanwhile, eight different monosaccharides were found in GSP-1-1 (Figure 5c), predominantly comprising galacturonic acid, arabinose, rhamnose, and galactose, accounting for 44.55%, 22.84%, 12.71%, and 12.53% of the molar ratio, respectively. Small amounts of glucose, glucuronic acid, mannose, and xylose were found, accounting for 2.3%, 2.18%, 1.68%, and 1.21%. The experimental evidence revealed that polysaccharides with higher proportions of mannose, arabinose, and glucose effectively reduced the MDA levels while enhancing the glutathione peroxidase, catalase, and superoxide dismutase activities in streptozocin-induced diabetic mice, demonstrating antidiabetic effects [43]. Conversely, the polysaccharides dominated by galactose and glucose elevated glycolytic enzyme activity and reduced hepatic transaminase activity in streptozocin-induced mice [44]. Collectively, GSPs demonstrate potential hypoglycemic activity, which may stem from their complex monosaccharide composition and elevated contents of galacturonic acid, arabinose, and rhamnose [43].

### 3.7. SEM Analysis

**Figure 6:**
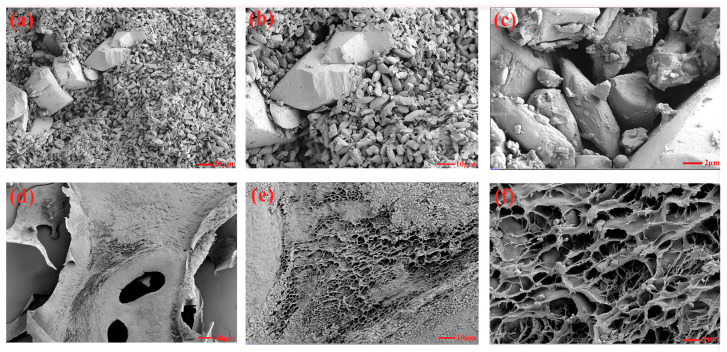
Structural morphology of GSPs (**a**–**c**) and GSP-1-1 (**d**–**f**).

Figure 6 provides the results of surface microstructure information of GSP and GSP-1-1, which was obtained under different magnifications by SEM. GSPs exhibited irregular block-like and rod-shaped morphologies, with a surface roughness characterized by distinct concavities and textural features, and GSP-1-1 had a cellular architecture composed of interconnected fibrous matrices. This structure contained heterogeneous pores with irregular geometries, accompanied by surface asperities. The interconnected porous structure of this polysaccharide provided it with an increased specific surface area and well-organized hierarchical porosity. These characteristics might improve the adsorption capacity of GSP-1-1, which can be used in the development of embedded materials [45,46]. Moreover, the spherical substructures on the reticular structure may promote the binding of GSPs to multiple targets, which is closely related to hypoglycemic effects [28].

### 3.8. Inhibition of α-Amylase and α-Glucosidase by GSPs

**Figure 7:**
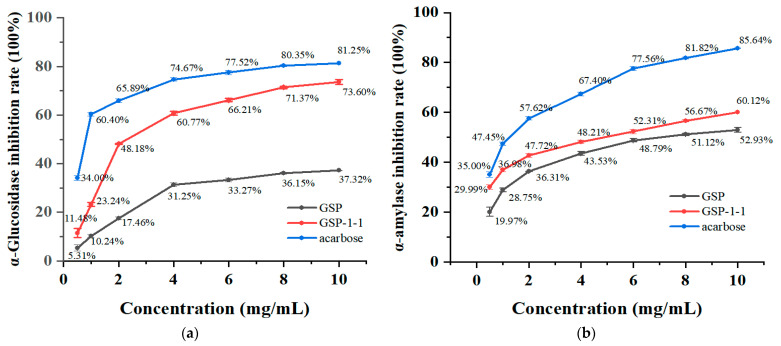
Inhibition rate of α-amylase (**a**) and α-glucosidase (**b**) by GSPs.

Figure 7a shows that GSP, GSP-1-1, and acarbose exhibited concentration-dependent inhibition (0–4 mg/mL) of α-glucosidase activity, with purified GSP-1-1 demonstrating superior efficacy. At 4 mg/mL, the inhibition rates reached 31.25%, 60.77%, and 74.67% for GSP, GSP-1, and acarbose, respectively, showing plateau tendencies. The corresponding CI50 values were determined as 2.39, 1.55, and 0.80 mg/mL for GSP, GSP-1-1, and acarbose. Compared with the polysaccharides of *Alhagi pseudalhagi* (7.15 mg/mL), the purified GSPs had a lower IC50 value, which means that high-purity GSPs have a more efficient glucosidase inhibition effect [47]. Previous studies by Ren et al. and Nie et al. have established a positive correlations between polysaccharide-mediated digestive enzyme inhibition (α-amylase/α-glucosidase) and uronic acid content [48,49,50].

The inhibitory effects of GSP, GSP-1-1, and acarbose on amylase activity were positively correlated with their concentrations (0.5–10 mg/mL). As illustrated in the Figure 7b, when the concentration was 10 mg/mL, the amylase inhibition rates reached 52.93%, 60.12%, and 85.64% for GSP, GSP-1-1, and acarbose, respectively, with a plateau in suppression efficiency. The IC50 values of GSP, GSP-1-1, and acarbose were determined to be 8.48, 4.27, and 2.27 mg/mL. Within the tested concentration range, GSP-1-1 exhibited slightly higher amylase inhibitory activity compared to GSP, although both were significantly weaker than acarbose. Compared with the polysaccharides of *Pithecellobium clypearia benth* (4.62 mg/mL), the purified GSPs had a lower IC50 value, which means that high-purity GSPs have the potential in the development of highly effective hypoglycemic drugs [28]. In summary, GSPs demonstrated significant inhibitory activity against α-glucosidase and α-amylase, highlighting their potential as therapeutic candidates for diabetes management, and the previous literature suggests that the enzyme inhibition of GSPs was competitive [51].

## 4. Discussion

Ultrasound-assisted extraction has emerged as an effective method for extracting bioactive compounds through modifications of polysaccharides’ chemical or steric configurations [52]. Furthermore, several research studies have demonstrated their viability for large-scale industrial applications [53,54]. This study optimized the ultrasound-assisted extraction process of GSPs by employing a BBD with three variables and three levels using the RSM. The optimal conditions comprised an ultrasonic temperature of 50 °C, an ultrasonic duration of 50 min, a liquid-to-solid ratio of 20 mL/g, and a sonic power of 210 W. Under these conditions, the extraction efficiency of GSPs was determined to be 11.92 ± 0.44%. Ultrasound-assisted extraction exhibited a dual role in polysaccharide processing: it simultaneously improved extraction yield and enabled controlled molecular degradation coupled with structural reconfiguration. This interplay enhanced the biofunctional properties, positioning ultrasound as a practically feasible approach for obtaining bioactive polysaccharides with tailored structural properties. A review of the existing literature indicated a low polysaccharide content in grape skins. Compared with previously reported extraction yields of polysaccharides from grape skins (8.02 ± 0.029), the method in this experiment has obvious advantages [3,16]. Nevertheless, parameter optimization remains critical to mitigate macromolecular degradation risks [55].

Polysaccharides exhibit diverse biological activities [53,56,57,58], which arise from their structural complexity, including heterogeneous molecular weight distributions and variations in monosaccharide composition. Crucially, molecular weight governs bioavailability by determining the ability of polysaccharides to traverse biological barriers and exert functional effects [59]. Furthermore, functional specificity is predominantly dictated by monosaccharide type [55]. GSPs were effectively purified into one component by different chromatographic columns, which were isolated as GSP-1-1 with a molecular mass of 101.856 kDa. The compositional analysis elucidated that GSP-1-1 consisted of galacturonic acid, arabinose, rhamnose, galactose, glucose, glucuronic acid, mannose, and xylose in a molar ratio of 40.26:26.99:13.58:12.20:2.24:1.97:1.63:1.42. In addition, GSP-1-1 exhibited greater inhibitory effects in terms of hypoglycemic activity, likely as a result of its higher uronic acid content. These findings substantiate the pivotal role of ultrasound-assisted extraction in isolating bioactive compounds, and this study proposes a novel strategy for valorizing the nutrient resources in grape byproducts. Further research will elucidate the hypoglycemic mechanisms of GSPs and their interplay with metabolic pathways.

## Figures and Tables

**Table 1:** The BBD factors and levels.

Level	Factor		
	Z_1_: Ultrasonic Time (min)	Z_2_: Ultrasonic Temperature (°C)	Z_3_: Liquid-to-Solid Ratio (mL/g)
−1	45	45	15
0	50	50	20
1	55	55	25

**Table 2:** Box–Behnken test results for GSP.

Run	Factor			Yield Rate (%)
	Z_1_	Z_2_	Z_3_	
1	−1	1	0	9.27 ± 0.01
2	0	0	0	11.69 ± 0.10
3	0	1	−1	8.73 ± 0.03
4	0	0	0	11.98 ± 0.01
5	1	−1	0	8.70 ± 0.03
6	0	0	0	11.82 ± 0.09
7	1	1	0	8.97 ± 0.02
8	0	−1	−1	8.50 ± 0.01
9	0	1	1	9.43 ± 0.02
10	1	0	−1	8.63 ± 0.01
11	1	0	1	10.30 ± 0.01
12	−1	−1	0	7.93 ± 0.01
13	0	0	0	12.04 ± 0.10
14	0	0	0	11.68 ± 0.07
15	−1	0	1	9.28 ± 0.05
16	0	−1	1	8.93 ± 0.04
17	−1	0	−1	8.63 ± 0.01

**Table 3:** ANOVA results for GSPs.

Source	Sum of Squares	DF	Mean Square	F-Value	*p*-Value	Significance
Model	33.1700	9	3.6900	62.2900	<0.0001	**
Z_1_	0.2742	1	0.2742	4.6300	0.0684	-
Z_2_	0.6827	1	0.6827	11.5400	0.0100	*
Z_3_	1.4800	1	1.4800	1.0500	0.0016	*
Z_1_Z_2_	0.2846	1	0.2846	4.8100	0.0644	-
Z_1_Z_3_	0.1700	1	0.1700	4.3400	0.0756	-
Z_2_Z_3_	0.0182	1	0.0182	0.3080	0.5962	-
Z_1_^2^	8.3300	1	8.3300	140.7300	<0.0001	**
Z_2_^2^	12.4400	1	12.4400	210.2200	<0.0001	**
Z_3_^2^	6.3100	1	6.3100	106.6200	<0.0001	**
Residual	0.4142	7	0.0592			
Lack of Fit	0.3062	3	0.1021	3.7800	0.1159	-
Pure Error	0.1081	4	0.0270			
Total	33.5900	16				

R^2^ = 0.9877; adjusted R^2^ = 0.9818; predicted R^2^ = 0.8491; adeq precision = 20.7383; - = not significant; ** = extremely significant; * = significant; coefficient of the variation (C.V.%) = 2.48.

**Table 4:** Chemical composition analysis of GSPs.

Item	Carbohydrate (%)	Protein (%)	Uronic Acid (%)	Sulfuric Radical (%)
GSP	37.68 ± 1.67%	1.69 ± 0.23%	4.26 ± 0.20%	5.87 ± 0.11%
GSP-1-1	87.79 ± 0.51%	-	9.31 ± 0.15%	0.67 ± 0.05%

-, Not detected.

## Data Availability

The original contributions presented in the study are included in the article. Further inquiries can be directed to the corresponding author.
